# Seed/Catalyst-Free Growth of Gallium-Based Compound Materials on Graphene on Insulator by Electrochemical Deposition at Room Temperature

**DOI:** 10.1186/s11671-015-0943-y

**Published:** 2015-05-27

**Authors:** Freddawati Rashiddy Wong, Amgad Ahmed Ali, Kanji Yasui, Abdul Manaf Hashim

**Affiliations:** Malaysia-Japan International Institute of Technology, Universiti Teknologi Malaysia, Jalan Sultan Yahya Petra, 54100 Kuala Lumpur, Malaysia; Department of Electrical Engineering, Nagaoka University of Technology, Kamitomioka-machi, Nagaoka, Niigata 940-2137 Japan

**Keywords:** Nanostructure, Thin film, Electrochemical deposition, Graphene, Gallium nitride, Gallium oxide

## Abstract

We report the growth of gallium-based compounds, i.e., gallium oxynitride (GaON) and gallium oxide (Ga_2_O_3_) on multilayer graphene (MLG) on insulator using a mixture of ammonium nitrate (NH_4_NO_3_) and gallium nitrate (Ga(NO_3_)_3_) by electrochemical deposition (ECD) method at room temperature (RT) for the first time. The controlling parameters of current density and electrolyte molarity were found to greatly influence the properties of the grown structures. The thicknesses of the deposited structures increase with the current density since it increases the chemical reaction rates. The layers grown at low molarities of both solutions basically show grain-like layer with cracking structures and dominated by both Ga_2_O_3_ and GaON. Such cracking structures seem to diminish with the increases of molarities of one of the solutions. It is speculated that the increase of current density and ions in the solutions helps to promote the growth at the area with uneven thicknesses of graphene. When the molarity of Ga(NO_3_)_3_ is increased while keeping the molarity of NH_4_NO_3_ at the lowest value of 2.5 M, the grown structures are basically dominated by the Ga_2_O_3_ structure. On the other hand, when the molarity of NH_4_NO_3_ is increased while keeping the molarity of Ga(NO_3_)_3_ at the lowest value of 0.8 M, the GaON structure seems to dominate where their cubic and hexagonal arrangements are coexisting. It was found that when the molarities of Ga(NO_3_)_3_ are at the high level of 7.5 M, the grown structures tend to be dominated by Ga_2_O_3_ even though the molarity of NH_4_NO_3_ is made equal or higher than the molarity of Ga(NO_3_)_3_. When the grown structure is dominated by the Ga_2_O_3_ structure, the deposition process became slow or unstable, resulting to the formation of thin layer. When the molarity of Ga(NO_3_)_3_ is increased to 15 M, the nanocluster-like structures were formed instead of continuous thin film structure. This study seems to successfully provide the conditions in growing either GaON-dominated or Ga_2_O_3_-dominated structure by a simple and low-cost ECD. The next possible routes to convert the grown GaON-dominated structure to either single-crystalline GaN or Ga_2_O_3_ as well as Ga_2_O_3_-dominated structure to single-crystalline Ga_2_O_3_ structure have been discussed.

## Background

The performance of silicon ultra-large-scale integrated circuits (Si-ULSIs) has been enhanced over the last 30 years by increasing the number of transistors in accordance with Moore’s law [1]. The scaling rule of the Si transistor has made it possible to miniaturize the transistors in the Si-ULSIs. However, the miniaturization of the transistors becomes increasingly difficult owing to the physical limitations, and the conventional scaling rule will not be enough to enhance the performance of the Si-ULSIs. Recently, the concept of advanced heterogeneous integration on Si platform was proposed towards the realization of a so-called “More than Moore” technology [2]. Here, semiconductor materials with superior properties are introduced on the Si platform in order to not only enhance the performance of MOS transistors [3] but also facilitate the present Si-ULSIs with various functionalities where these materials can be used to fabricate various kinds of functional devices, such as optical devices [4], photodetectors [5], solar batteries [6], and so forth. As a next-generation technology, such intelligent system-on-chip (i-SoC) on Si is considered as a promising and practical direction. However, in order to be able to fabricate electronic devices in those semiconductor materials, it is necessary to electronically isolate such materials and the Si substrate by the conventional insulators such as silicon dioxide (SiO_2_) or silicon nitride (Si_3_N_4_). Therefore, some breakthrough on growth technologies is strongly required to realize high-quality semiconductor-on-insulator on Si structures.

Gallium (Ga)-based compound materials such as gallium oxynitride (GaON) [7–9], gallium nitride (GaN) [10], and gallium oxide (Ga_2_O_3_) [11] are among the promising inorganic compound semiconductors that provide many advantages over other organic materials for electronic and optoelectronic device applications [12–19]. Graphene, a carbon allotrope, possesses high carrier mobility, exceeding 10^4^ cm^2^/Vs, even at room temperature (RT) [20]. The quantum Hall effect exists in graphene at RT, owing to ballistic transport of electrons and holes [21], and this means that graphene is potentially useful for ballistic device applications [22]. Graphene has also been shown as a material with high thermal conductivity of 10^3^ W/mK [23–26]. It is also well documented that graphene has a great potential for novel electronic devices to act as transparent electrode [27], sensing membrane [28], and so forth, because of its extraordinary electrical, thermal, and mechanical properties.

Since the conventional insulators, i.e., SiO_2_, Si_3_N_4_, are amorphous or polycrystalline, the resulting grown semiconductor structures on top are normally going to be also amorphous or polycrystalline. However, such problem can be solved by utilizing graphene sheets as a template layer. The bonding structure of graphene is also similar to the *c*-plane of a hexagonal crystalline structure and (111) plane of zinc-blende structure. With this regard, the growth of Ga-based compound materials on graphene seems to be feasible. Recently, we have demonstrated the growth of several kinds of materials such as germanium (Ge) thin film [29], silicon carbide (SiC) thin film [30], and zinc oxide (ZnO) nanostructures [31–35] with considerably good properties directly on graphene without any use of seed or catalyst.

It is worth noting that a hybrid structure of semiconductor nanostructures and thin films on graphene is particularly interesting because these structures can offer additional functionality and flexibility for realizing advanced electronic and optoelectronic applications in photovoltaics, nanogenerators, field emission devices, sensitive biological and chemical sensors, and efficient energy conversion and storage devices [36–40]. Thus, with the excellent electrical and thermal characteristics of graphene, growing semiconductor nanostructures and thin films on graphene layers would enable their novel physical properties to be exploited in diverse sophisticated device applications. In addition, graphene which is formed by the weakly bonded layers of two-dimensional hexagonally arranged carbon atoms held together by strong triangular covalent σ-bonds of the sp^2^-hybridized orbitals can allow the transfer of the grown inorganic nanostructures or films onto the other arbitrary substrates such as glass, metal, and plastic easily [38].

The most common method to grow inorganic semiconductors on graphene is vapor-phase technique such as metal-organic vapor-phase epitaxy (MOVPE) [41, 42]. For example, GaN has been successfully grown on the ZnO-coated [38, 42, 43] and AlN-coated graphene [44] by such kind of vapor-phase technique. However, the vapor-phase method is likely to involve high-temperature process and is also considered as a high-cost method. In this work, a liquid-phase method, namely an electrochemical deposition (ECD), is used. This electrochemical deposition seems to be a promising method to grow Ga-based inorganic semiconductors on graphene at room temperature with good controllability in terms of growth rates and structure dimensions [44].

Up to this date, there is no report on the seed/catalyst-free growth of such Ga-based compound materials on graphene by an electrochemical deposition technique. Recently, we reported the growth of Ga_2_O_3_ on silicon (Si) by an electrochemical deposition [45] and GaN nanostructures on Si by a nitridation of electrochemically deposited Ga_2_O_3_ [46]. In this paper, we report the direct growth of Ga-based compound materials on graphene on insulator by an electrochemical deposition. It was found that the grown structures were formed by Ga_2_O_3_ and GaON, and their properties are effectively controlled by the current density and molarity ratio of the electrolytes. The similarity of atomic arrangement of graphene with certain planes of hexagonal crystalline structure and zinc-blende structure enables the induction of epitaxial growth of Ga-based compounds on graphene with the assistance of the flows of charged ions generated in the electrodeposition process. It is worth noting that the growth of graphene is not considered in this study and commercially available transferred chemical vapor deposition (CVD) grown graphene on SiO_2_/Si substrate is used.

## Methods

**Fig. 1:**
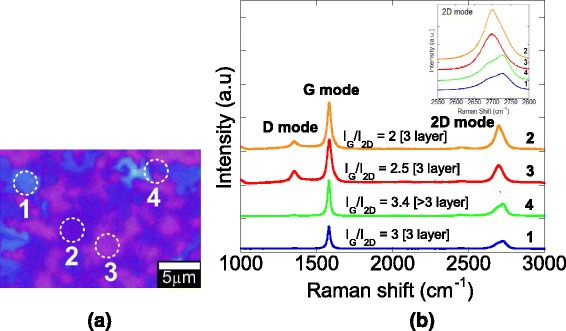
**a** Nomarski image of multilayer graphene and **b** Raman spectra of multilayer graphene

The commercially available CVD grown multilayer (ML) graphene on SiO_2_ (285 nm)/Si substrates (Graphene Laboratories Inc, Calverton, NY, USA) was used. The Nomarski optical image of ML graphene in Fig. 1a shows the visibility of graphene sheets on SiO_2_/Si substrate with a different number of layers which is consistent with the measured Raman spectra shown in Fig. 1b. It is worth noting that the applications of Raman spectroscopy are widely used to characterize graphitic materials. In graphene, the phonon energy shift caused by laser excitation creates three main peaks known as G band (peak: ~1580 cm^−1^), D band (peak: ~1350 cm^−1^), and 2D band (peak: ~2700 cm^−1^) [47]. The G band is an in-plane vibrational mode involving the sp^2^-hybridized carbon atoms that comprises the graphene sheet. The G band position is highly sensitive to the number of layers present in the sample and is one method for determining layer thickness. The D band is known as the disorder band or the defect band and it represents a ring breathing mode from sp^2^ carbon rings, although to be active, the ring must be adjacent to a graphene edge or a defect. The band is typically very weak in graphite and is typically weak in high-quality graphene as well. If the D band is significant, it means that there are a lot of defects in the material. The intensity of the D band is directly proportional to the level of defects in the sample. The 2D band is the second order of the D band, sometimes referred to as an overtone of the D band. It is the result of a two-phonon lattice vibrational process, but unlike the D band, it does not need to be activated by proximity to a defect. As a result, the 2D band is always a strong band in graphene even when there is no D band present, and it does not represent defects. This band is also used to determine graphene layer thickness. In contrast to the G band position method, the 2D band method depends not only on band position but also on band shape. Ferrari *et al.* reported that the 2D peaks which occur at ~2700 cm^−1^ for bulk graphite have much broader and up-shifted 2D band which can be correlated to few layer graphene [47]. D and 2D peak positions are dispersive depending on the laser excitation energy. Here, the sample is cited from a 514-nm excitation laser. Because of added forces from the interactions between layers of stacked multilayer graphene, as the number of graphene layers increases, the spectrum will change from that of single-layer graphene, namely a splitting of the 2D peak into an increasing number of modes that can combine to give a wider, shorter, and higher frequency peak. The G peak also experiences a smaller red shift from the increased number of layers. Thus, for stacked graphene, the number of layers can be derived from the ratio of peak intensities, *I*_G_/*I*_2D_, as well as the position and shape of these peaks [47]. The growth of Ga-based compounds on graphene/SiO_2_/Si was carried out by a cathodic electrochemical deposition in a mixture of ammonium nitrate (NH_4_NO_3,_ Sigma Aldrich, ≥98 % purity) and gallium nitrate (Ga(NO_3_)_3_, Sigma Aldrich, ≥99.9 % purity) dissolved in deionized (DI) water at room temperature. In our ECD system, platinum (Pt) wire acted as an anode (counter electrode) while graphene acted as a cathode. Graphene is not only used as the template but also it acts as an cathode to complete the circuit of the electrodeposition since the core structure of SiO_2_/Si does not allow the flow of current on its surface. Without any flow of charges through SiO_2_ surface, the deposition is not able to be induced on the surface.

Both anode and cathode were connected to the external direct current (DC) power supply. In this experiment, the electrodeposition was operated under galvanostatic control where the current density was fixed during the deposition. It is noted here that the distance between the two electrodes was fixed at 1 cm for all experiments in order to avoid the other possible effects apart from the current density. The deposition was performed at different current densities ranging from 0.5 to 3.5 mA/cm^2^ for 6 h. The molarities of NH_4_NO_3_ and Ga(NO_3_)_3_ solutions were varied from 2.5 to 15 M and 0.8 to 15 M, respectively.

After 6 h, the sample was removed immediately from the electrolyte and quickly rinsed with DI water to remove any residue from the surface. The surface morphology, elemental composition, crystallinity, and elemental bonding properties of the grown Ga-based compounds were characterized using field emission scanning electron microscopy (FESEM; Hitachi SU8083), energy dispersive X-ray (EDX) spectroscopy, X-ray diffractometer (XRD; Bruker D8 Advance), and Fourier transform infrared spectroscopy (FTIR; Agilent Technologies Cary 600 Series).

## Results and Discussion

First, the chemical reactions that are expected to take place during the growth need to be formulated in order to predict the possible grown structures. In this work, Ga(NO_3_)_3_ and NH_4_NO_3_ are used as Ga and N source, respectively, to form GaN-related structures. However, as described in the following section, the existence of H_2_O in the solutions may generate excessive O atoms, which in turns might lead to the formation of GaON and Ga_2_O_3_. The details of possible chemical reactions involved can be described as the following:

NH_4_NO_3_ → NH^4+^ + NO^3−^ (1)

NH^4+^ + NO^3−^ → NH_3_ + HNO_3_ (2)

Ga(NO_3_)_3_·H_2_O → Ga^3+^ + 3NO^3−^ + H_2_O (3)

Ga(NO_3_)_3_ + NH_3_ → GaON + HNO_3_ + H_2_ + 2NO_2_ + O_2_ (4)

Ga^3+^ + 2H_2_O → GaOOH + 3H^+^ (5)

2GaOOH → Ga_2_O_3_ + H_2_O (6)

Here, at the initial step of reaction, both NH_4_NO_3_ and Ga(NO_3_)_3_ will be ionized. The ionization of NH_4_NO_3_ will produce NH^4+^ and NO^3−^ ions. Furthermore, the NH^4+^ ion will donate its most acidic proton, and hence, NH_3_ and HNO_3_ will be produced. On the other hand, the ionization of Ga(NO_3_)_3_ will result in the production of Ga^3+^, NO^3−^ ions, and water. A rapid reaction will take place between Ga^3+^ ions and water molecules to produce GaOOH and hydrogen protons through the hydrolysis. Such intermediate reaction might proceed to produce Ga_2_O_3_. Due to the presence of the NH_3_ resulted from the ionization of the NH_4_NO_3_, the production of GaON is expected to result from the reaction of the NH_3_ and excessive Ga(NO_3_)_3_ in an oxygen rich environment. From these proposed reactions, it seems to suggest that the grown structures could be controlled to be GaON-dominated as compared to Ga_2_O_3_-dominated structures at the conditions where the molarity of Ga(NO_3_)_3_ is kept at a low value and the molarity of NH_4_NO_3_ at a high value. On the other hand, Ga_2_O_3_-dominated structures seem to be obtainable when the molarity of Ga(NO_3_)_3_ is kept at a high value and the molarity of NH_4_NO_3_ at a low value. The above reactions continuously take place and lead to the growth of thin film. In conclusion, it can be understood that the following two structures are possible to be grown: i) structure with a mixture of both Ga_2_O_3_ and GaON and ii) structure with either Ga_2_O_3_ or GaON. The properties of the structure could be optimized by the main control parameters of the current densities and molarities of solutions. Therefore, this work is going to investigate the effects of the current densities and molarities of solutions on the grown structures.

**Fig. 2:**
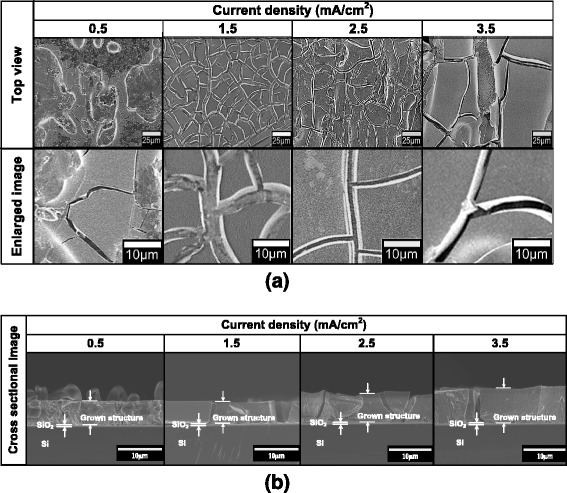
**a** Top view and **b** cross-sectional view of FESEM images of Ga-based compound structures grown at current densities of 0.5, 1.5, 2.5, and 3.5 mA/cm^2^. NH_4_NO_3_ = 2.5 M, Ga(NO_3_)_3_ = 0.8 M

At first, we investigate the effects of current densities on the grown structures by keeping the molarities of solution to be constant. Fig. 2a shows the FESEM images of the grown structures at current densities of 0.5, 1.5, 2.5, and 3.5 mA/cm^2^, respectively. Here, the molarities of Ga(NO_3_)_3_ and NH_4_NO_3_ were fixed at 0.8 and 2.5 M, respectively. Such values of molarities of solutions are chosen based on the reported work by Al-Heuseen *et al.* [48, 49]. They studied the electrodeposition of GaN on Si (111) substrate using the same mixture of electrolyte and range of current densities. However, they had only succeeded in depositing a network of nanoflake structures at a very long time of 12 to 48 h, and the structures contain the hexagonal (h-) and cubic (c-) phases of GaN together with beta (β-) phase of Ga_2_O_3_.

As shown in Fig. 2a, all grown structures show non-continuous film structures with grain-like morphologies surrounded with wide cracking structures. Recently, we have also observed the same tendency for the growth of SiC on single-layer graphene (SLG) where grain-like film structure was obtained [30]. However, in the case of the growth of SiC on graphene, the grain-like film structures are continuous without any cracking structure. As reported in ref. [30], the resulted grain structures are presumably due to the nature of grain-like structure of polycrystalline graphene used. The possible reason why the cracking is generated in this growth is speculated due to the stacking structures or uneven thicknesses of MLG [34]. From the cross-sectional FESEM images shown in Fig. 2b, it can be clearly seen that the sizes and the thicknesses of deposited grain structures increase with the current densities. As can be seen in Fig. 2b, the thicknesses of deposited grain layer increase from 4.3 μm at current density of 0.5 mA/cm^2^ to 6.1 μm at current density of 3.5 mA/cm^2^. Also, the width of cracking structures also seems to increase with the current densities. In principle, we believe that the morphology of the grown structures is determined by the nature of grain-like structure of polycrystalline MLG. When the current densities are increased, the deposition rates of the grown layers increase resulting to the increase in the thicknesses. Such high deposition rates seem to be able to combine the small grain-like structures by covering the grain boundaries especially at the grain area with same graphene thicknesses or even with small difference of thicknesses. Thus, such mechanism leads to the formation of larger grain-like structures. The cracking structures are still being observed and not able to be eliminated presumably due to the large difference in the graphene thicknesses. The cracking structure becomes apparent due to thick deposited layer. Meanwhile, the EDX spectra (data not shown) confirmed that the grown films contain Ga, O, and N elements indicating the possible formation of GaON and Ga_2_O_3_ structures.

**Fig. 3:**
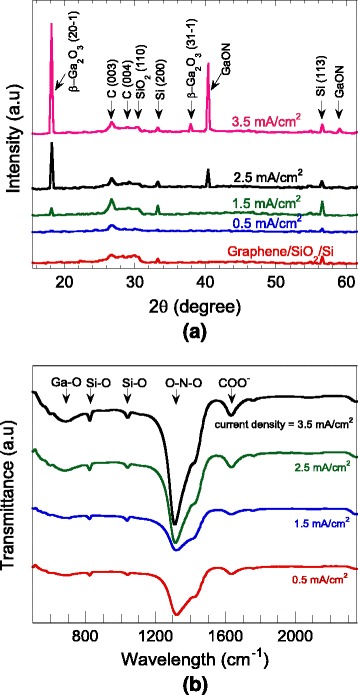
**a** XRD spectrum and **b** FTIR transmission spectrum of the synthesized Ga-based compound materials at current densities of 0.5, 1.5, 2.5, and 3.5 mA/cm^2^. NH_4_NO_3_ = 2.5 M, Ga(NO_3_)_3_ = 0.8 M

Figure 3a shows the XRD spectrum of the above corresponding as-deposited grown structures together with bare MLG/SiO_2_/Si substrate for comparison. The intensities found at 2*θ* values equal to 18.24° and 38° are attributed to β-Ga_2_O_3_ in (20-1) and (31-1) planes, respectively (ICDD: 01-074-1776) which were found to be very small at low current density but increase significantly with the increase of the current densities. The same tendency is also observed for the intensities of peaks at 2*θ* values equal to 40.4° and 58.8° which are ascribed to GaON based on the reported work by Cailleaux *et al*. [7]. It was reported that a peak at 40.4° can be indexed to cubic (sphalerite-type) structure of GaON unit cell, while a peak of 58.8° can be indexed to hexagonal (wurtzite-type) structure of GaON unit cell. Here, GaON could be considered as a polytype in which the cubic and hexagonal arrangements are coexisting [7]. The increases in the intensities of the peaks for both Ga_2_O_3_ and GaON simply indicate the increase in thicknesses of the grown structures. It is speculated that GaON was grown by the introduction of O species into the vacancies of the Ga-site lattice, where a possible side reaction may proceed as follows: Ga_2_O_3_ + GaN → Ga_*x*_O_*y*_N, where *x* and *y* refer to the atomic % of both elements in the reaction products. As a conclusion, it can be said that high current density is needed in promoting the chemical reactions to form Ga-based compounds and the formation of both Ga_2_O_3_ and GaON seems to increase in the similar rate with the current density. From the XRD spectra, we can assume that the grown structures are basically polycrystalline due the mixture of Ga_2_O_3_ and GaON structures. Also, as mentioned above, GaON is well known to exist as a polytype in which the cubic and hexagonal arrangements are coexisting.

Figure 3b shows the FTIR spectrum of the corresponding grown structures. Five significant band peaks at 680, 826, 1040, 1322, and 1633 cm^−1^ were observed. Among them, two band peaks corresponding to 826 and 1040 cm^−1^ can be attributed to the bands of Si-O stretching mode [50, 51]. Meanwhile, the band peaks at 680 and 1322 cm^−1^ are attributed to the local vibrational mode of Ga-O bond [52] and symmetric stretching of O-N-O band, respectively [53, 54]. The O-N-O band suggests the formation of GaON clusters around N centers. Another band peak at 1633 cm^−1^ can be attributed to the bond of the carboxylic group that belongs to the graphene structure [55]. The intensities of the valley peaks of Ga-O and O-N-O band increase with the current densities which indicate the increase of Ga-O and Ga-O-N bonds in the grown structures. The presented results of FTIR, FESEM, and XRD so far are consistent to each other.

**Fig. 4:**
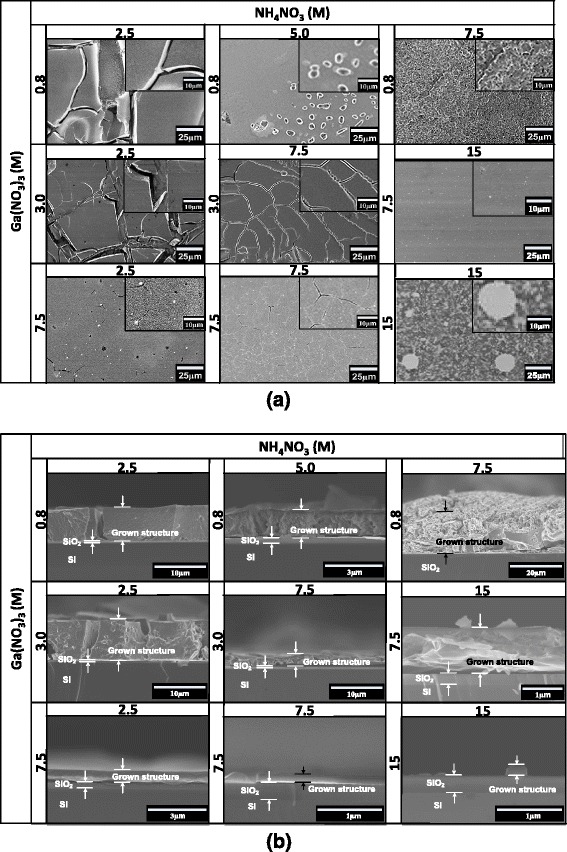
**a** Top view and **b** cross-sectional view of FESEM images of the synthesized Ga-based compound structures grown at various combination of solution molarities. Current density = 3.5 mA/cm^2^

In the next study, we investigate the effects of molarities of Ga(NO_3_)_3_ and NH_4_NO_3_ on the grown structures. Here, the fixed current density of 3.5 mA/cm^2^ was selected since such high current density is found to be favorable to increase the chemical reactions or thicknesses. Fig. 4a summarizes the FESEM images of the grown structures with the changes in the combination of the molarities of Ga(NO_3_)_3_ and NH_4_NO_3_. It can be seen that the structures grown at low molarities basically show cracking structures and such structures seem to diminish with the increases of molarities of solutions except molarity combination of 15 M for both solutions. The structures with uniform and continuous layers with less cracking structures are realized at the molarity combination of 7.5 and 15 M of Ga(NO_3_)_3_ and NH_4_NO_3_, respectively. It is speculated that the increase of ions in the solutions helps to promote the growth at the area with uneven thicknesses of graphene. The highest thickness of deposited film with less cracks was 16.7 μm, grown at the lowest molarity of Ga(NO_3_)_3_, 0.8 M, and high molarity of NH_4_NO_3_, 7.5 M as shown in Fig. 4b. It can be seen in Fig. 4b that the layers grown at high molarity of Ga(NO_3_)_3_ tend to be relatively thin. It can be concluded that to produce thick layer, low molarity of Ga(NO_3_)_3_ and high molarity of NH_4_NO_3_ are needed. While, high molarity of Ga(NO_3_)_3_ of over 7.5 M seems to slow down the deposition process. Moreover, when the molarity of Ga(NO_3_)_3_ is increased to 15 M, the cluster-like structure was formed instead of film-like structure as shown in Fig. 4b.

**Fig. 5:**
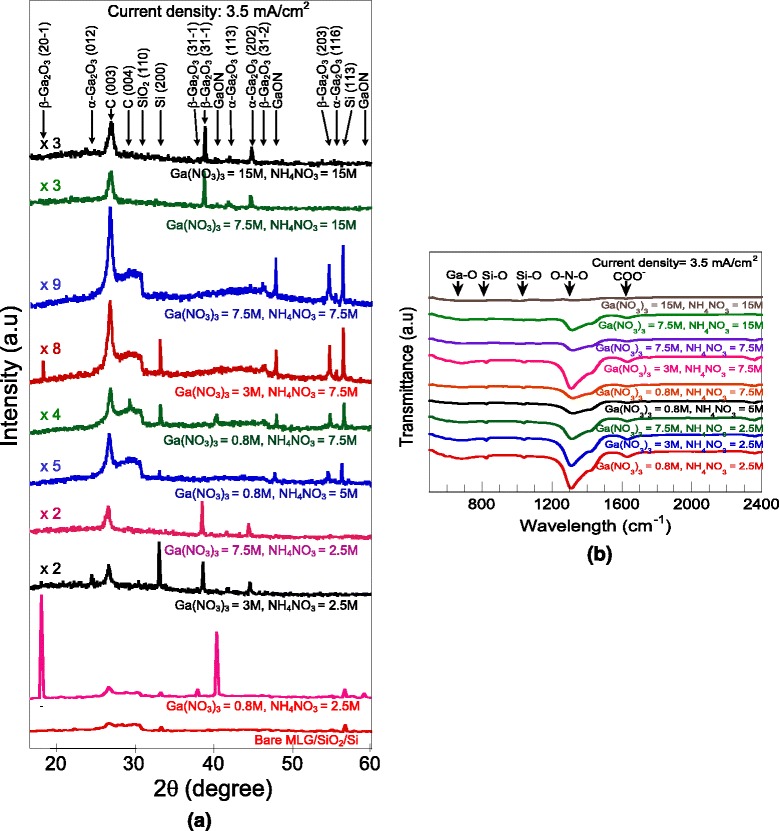
**a** XRD spectrum and **b** FTIR transmission spectrum of the synthesized Ga-based compound materials at various combination of solution molarities. Current density = 3.5 mA/cm^2^

Figure 5a shows the XRD spectrum of the corresponding as-deposited structures and bare MLG/SiO_2_/Si substrate for comparison. As shown in Fig. 5a, a mixture of Ga_2_O_3_ and GaON structures was observed in sample grown at the lowest molarities of both solutions, i.e., 0.8 M for Ga(NO_3_)_3_ and 2.5 M for NH_4_NO_3_. When the molarity of Ga(NO_3_)_3_ is increased while keeping the molarity of NH_4_NO_3_ at the lowest value of 2.5 M, the grown structures are basically dominated by the Ga_2_O_3_ structure since only Ga_2_O_3_-related peak is observed without any GaON-related peak. This is due to the increase of Ga^3+^ ions to react with water to form GaOOH which finally dehydrate to form Ga_2_O_3_. On the other hand, when the molarity of NH_4_NO_3_ is increased while keeping the molarity of Ga(NO_3_)_3_ at the lowest value of 0.8 M, the GaON peaks appear. However, Ga_2_O_3_-related peak with low intensity is still being detected in the grown structures suggesting a mixture of small portion of Ga_2_O_3_ in the grown structures. GaON is formed due to the increase of NH_3_ molecules supplied from NH_4_NO_3_ to intensively react with the Ga(NO_3_)_3_. It is speculated that the gallium vacancies induced by the substitution of nitride ions with oxide ions forming GaON. The small portion of Ga_2_O_3_ in the grown structures seems to be unavoidable due to the reaction of Ga^3+^ and water persists during the growth. Here, it is speculated that GaON-dominated structure may be produced if the molarity of Ga(NO_3_)_3_ is further decreased below 0.8 M. When the molarities of Ga(NO_3_)_3_ are further increased to the level of 7.5 M, the grown structures tend to be dominated by Ga_2_O_3_ even though the molarity of NH_4_NO_3_ is made equal or higher than the molarity of Ga(NO_3_)_3_ as shown by the XRD spectra. Here, it is worth noting that the thicknesses of the structures grown at such molarity of Ga(NO_3_)_3_ are relatively thin which is below 0.7 μm. Al-Husseen *et al*. also observed that when the grown structure is dominated by the Ga_2_O_3_ structure, the deposition process became slow or unstable [48].

Figure 5b shows the FTIR spectrum of the above corresponding grown structures. Again, five significant band peaks at 680, 826, 1040, 1322, and 1633 cm^−1^ that are same with the spectrum shown in Fig. 3b were observed. Two band peaks corresponding to 826 and 1040 cm^−1^ are attributed to the bands of Si-O stretching mode [50, 51]. Meanwhile, the band peaks at 680 and 1322 cm^−1^ are attributed to the local vibrational mode of Ga-O bond [52] and symmetric stretching of O-N-O band, respectively [53, 54]. Here, it is noted that the Ga-O valley peak is too weak and not clearly observed. Also, another band peak at around 1633 cm^−1^ can be attributed to the bond of carboxylic group that resulted from graphene [55]. In consistent with the XRD results, it can be seen that the intensities of O-N-O band valley peak significantly decrease with the increase of the molarity of Ga(NO_3_)_3_ while keeping the molarity of NH_4_NO_3_ at the lowest value of 2.5 M. The formation of GaON can be obtained when the molarities of NH_4_NO_3_ is increased while keeping the molarity of Ga(NO_3_)_3_ at the lowest value of 0.8–3.0 M. Here, it can be also seen that at the high molarity of Ga(NO_3_)_3_ over 7.5 M, the formation of Ga-N is hardly observed especially at molarity of 15 M of Ga(NO_3_)_3_ where almost no O-N-O band valley peak is observed, suggesting the high domination of Ga-O in the grown structure. From these results, again, it is speculated that the formation of GaON can be promoted with further reduction of the molarity of Ga(NO_3_)_3_ and keeping the molarity of NH_4_NO_3_ at high values. It is worth noting that the intensities of both XRD and FTIR reflect to the thicknesses where the intensities increase with the thicknesses. The obtained results also show that the thicknesses of the grown structures strongly depended on the current densities and the combination of solution molarities.

Finally, as a brief remark, the next possible works are discussed. In recent years, a transformation of the grown gallium oxide (Ga_2_O_3_) structures to GaN by a so-called nitridation seems to be a simple method to create high quality of GaN structure [56]. Here, a nitridation is believed to be achievable by simply annealing the Ga_2_O_3_ structures in ammonia gas. Li *et al.* reported the repeatable transformation of the CVD grown GaN structures to Ga_2_O_3_ structures by an annealing in air and back to GaN structures by an annealing in ammonia [57]. Besides that, it was reported that surface treatment of GaON film with H_2_ at elevated temperature is also able to lead to the reduction of GaON into GaN [58]. Also, the grown GaON can be converted to Ga_2_O_3_ by simple annealing in oxygen as well. In conclusion, this study seems to successfully provide the conditions in growing either Ga_2_O_3_-dominated or GaON-dominated structure by a simple and low-cost ECD. Here, it is also proposed that such dominated structures could be easily converted to either single-crystalline GaN or Ga_2_O_3_ structures by using the abovementioned possible routes.

## Conclusions

The growth of Ga_2_O_3_ and GaON on insulator by utilizing graphene as template layer was achieved. The thicknesses of the deposited structures increase with the current density since it increases the chemical reaction rates. The selective growth is achievable by manipulating the molarity of the electrolytes towards either Ga_2_O_3_-dominated or GaON-dominated structures even by using a simple and low-cost ECD technique. The possible routes to convert the grown GaON-dominated structure to either single-crystalline GaN or Ga_2_O_3_ as well as Ga_2_O_3_-dominated structure to single-crystalline Ga_2_O_3_ structure have been briefly highlighted.
